# On the Treatment of Nasal Polypi

**Published:** 1892-04-23

**Authors:** 


### SURGERY.

### ON THE TREATMENT OF NASAL POLYPI.

There is no such thing as finality in medicine. So far from such an ideal state ever being arrived at on almost any single subject it would seem that whenever we appear to have reached a mode of treatment which is in every way satisfactory, fresh observations have shown that we have just touched the fringe of the question at issue. Not so many years ago the removal of benign nasal polypi was regarded as a simple matter of avulsion, and the descriptions given in text books would certainly lead one to regard the operation as one about which there should be little difficulty in the majority of cases. But the nose no longer enjoys the happy reputation it once had, and is now regarded with just suspicion in a great many respiratory affections, and it has been abundantly proved to originate a large proportion of asthmatic cases.

Dr. Greville Macdonald, for purposes of clinical expediency, divides the subject of nasal polypus into four clinical varieties, viz., (1) mucous, (2) cystic, (3) fibrous, and (4) cauliflower. In such a classification, he remarks, it must be clearly understood that pathology is not the basis, and that malignant and semi-malignant neoplasms, such as epithelioma and sarcoma, true naso-pharyngial fibroma and fibromucous polypi of the post-nasal space are excluded.

By far the most common variety is the mucous polypus. They are generally attached to the middle turbinated bone, and may grow from its inner or outer surface or its free border. Sometimes the polypus is attached to the superior turbinated bone, but never to the inferior turbinated, nor to the septum.

The growth is apparently started by a diseased condition of the mucous membrane, which in turn has resulted from a pathological condition of the bone underlying it. All authorities appear to be convinced of the paramount importance of treating the actual seat of attachment.

In a valuable contribution by Dr. W. E. Casselberry,* of Chicago, some of the difficulties in reaching the seat of attachment are discussed. The author directs attention to Zuckerkandl's researches on cadavers, which demonstrated that two-thirds of all nasal myxomata originated from the middle meatus, beneath the middle turbinated body, and that approximately two-thirds of this number took origin from the edges of the hiatus semilunaris, a numerical deduction which harmonises with Dr. Casselberry's own observations.

Now the hiatus semilunaris opens into the infundibular space, whose upward and downward continuations enter respectively the frontal and maxilary sinuses, and which is located high up beneath the middle turbinated body. Besides the hiatus semilunaris, the bulla ethmoidalis just above it not infrequently forms the seat of origin of polypi. The importance of radical surgical procedures for the establishment of a free nasal passage for respiration, drainage, vision, and instrumental manipulation have been urged by the author in a previous communication, and is, in fact, generally admitted. To this end the reduction of hypertrophied turbinated structures by the electro-cautery, the removal of septal excrescences by the saw, chisel, or motor drills, correction of septal deflection, and excision of hypertrophied tonsils or adenoids, may become necessary.

The real success of the treatment of polypi, after having gained access to them, consists in tracing them to their points of attachment and thoroughly cauterising these so-called roots if not at the same sitting, then at the next, remembering meanwhile the exact spot. Knowing the hiatus semilunaris to be a favourite point of origin, those polypi which proceed from beneath the middle turbinated body could be followed up by insinuating a fine electrode, slightly curved on the flat to this point; and those which spring from the meatus superior posteriorly must be reached by a properly curved point-electrode introduced through the mouth and naso-pharynx always under the best illumination.

The author does not mean by this that any considerable surface area of mucous membrane should be seared over, but that, as nearly as possible, exact spots of origin should be subjected to circumscribed but deep cauterisation. Also, when reducing hypertrophy of any of the turbinated bodies, but little surface area need be damaged by his method of making narrow but deep linear cauterisations by means of a point-electrode slightly curved on the flat. By this means, after removal by the snare, supplemented by an occasional use of the forceps, the majority of cases can be permanently cured, but a minority will still continue unrecovered.

But a certain proportion of cases, it would seem, cannot be effectually treated by these measures, and Dr. Casselberry's experience has convinced him that these exceptional cases are curable only after removal of the antero-inferior part of the middle turbinated body. The cases referred to are those in which the superior nasal zone is very narrow, and the turbinated body in contact with the septum and close also to the outer wall, in which cauterisation will only be followed by adhesions, with further obstruction to drainage. Moreover, the space is insufficient through which to insert an electrode to the region of the hiatus semilunaris; also when the bone of the turbinated body is itself enlarged or malformed it presents like impediment to access and treatment. The removal of the anterior-inferior end of the body then suffices to provide space for the passage of instruments to the seat of attachment for eradication of the polypi, to afford a proper drainage channel preventing accumulation of muco-purulent material, and to relieve pressure upon the venous escape, and œdema, thus guarding against recurrence. The turgid, swollen, water-soaked aspect of the membrane rapidly assumes a healthier appearance, and the inferior turbinates, when previously congested, will retract as a result of subsidence of irritation above. The size of the piece to be abscised is determined by individual circumstances.

The question next arises, Is the operation described justifiable to the end attained? Opinions differ. The middle turbinated body as a process of the ethmoid bone should, of course, be carefully dealt with, as the possibility of septic or other inflammation extending thence to the meninges cannot be denied, although the author is unfamiliar with testimony adequate to establish such an occurrence solely as a result of reasonably skilful operations.

For operating, Dr. Casselberry considers properly-constructed turbinate bone scissors the easiest and best means of abscission, provided space on each side of the body will permit the passage of blades sufficiently high up—the best means because a clean cut of the bone is made at the point selected, without risk of fracture at other points, as would seem possible by the crunching movement of a snare or the breaking action of forceps. He recommends as most useful in his experience the scissors with curved blades, with the edges serrated so as to prevent slipping. When the scissors cannot be manipulated, the snare will usually effect the purpose. The great disadvantage of the snare is the liability of the loop to slip and remove less than desired. Forceps must be used when, by reason of extreme narrowness or crowding of the parts, neither turbinate scissors nor snare can be satisfactorily manipulated.

That opinions should differ as to the advisability or necessity of such a procedure as that advocated by Dr. Casselberry is natural; but every one who has had a large experience in the treatment of polypi must have found great difficulty in dealing with the class of cases for which the operation is suggested.

Many other methods of treating the pedicle, and even the whole polypus, find favour with rhinologists of repute. In the discussion following the above-quoted communication, Dr. Solis Cohen bore testimony to the value of washing out the nose with a solution of alcohol or of witch-hazel. He uses it in the strength of one to four, one to three, or even one to two—simply distilled extract of hamamelis with alcohol. Dr. Bosworth had, however, found it useless.

Dr. Jarvis has found that the injection of the chloride of iron into the nostril will cause polypi to disappear, but recommends the application of the solution to the seat of the pedicle after removal of the polypus.

We cannot too strongly emphasise the importance of

* *New York Med. Jour.*, vol. liv., p. 533.

guiding the hand by the eye. To simply push in a polypus forceps, seize whatever feels like the polypi, give several turns to the instrument till whatever has been seized comes away, and when, perhaps, after a few unsuccessful "shots" a polype appears, to fancy one has done all that can be done for our patient, is altogether unworthy of the name of surgery.

